# Graphene Oxide (GO) and Gold Nanoparticles (AuNP) Facilitated Electrochemical Biosensing for Lung Cancer Diagnosis

**DOI:** 10.3390/diagnostics16142179

**Published:** 2026-07-13

**Authors:** Rekerayi Chibagidi, Palesa Pamela Seele, Valentine Saasa

**Affiliations:** 1 Department of Life and Consumer Sciences, College of Agriculture and Environmental Sciences, University of South Africa (UNISA), Florida Campus, Roodepoort 1709, South Africa; 25200038@mylife.unisa.ac.za; 2 Health Platform, Advanced Materials Division, Mintek, Randburg, Johannesburg 2025, South Africa; palesas@mintek.co.za; 3 Department of Science, Technology and Innovation/Mintek Nanotechnology Innovation Centre, Advanced Materials Division, Mintek, Randburg, Johannesburg 2025, South Africa

**Keywords:** electrochemical biosensors, gold nanoparticles, graphene oxide, lung cancer biomarkers, diagnostics

## Abstract

Early detection of lung cancer remains challenging due to the extremely low concentrations of disease-specific biomarkers, which limit the development of highly sensitive and reliable point-of-care (PoC) diagnostic devices. Electrochemical biosensors integrating graphene oxide (GO) and gold nanoparticles (AuNPs) have emerged as promising platforms for the rapid, sensitive, and selective detection of lung cancer biomarkers, enabling more timely diagnosis. Biomarkers such as carcinoembryonic antigen (CEA), cytokeratin-19 fragments (CYFRA 21-1), neuron-specific enolase (NSE), and circulating tumour DNA are increasingly investigated for PoC applications since they can be detected in various biological fluids associated with lung cancer. Nanocomposite materials, particularly GO/AuNP hybrids, provide synergistic advantages by combining the large surface area and abundant functional groups of GO for stable immobilization of biorecognition elements with the excellent conductivity and bioconjugation capability of AuNPs that enhance signal transduction. This review critically discusses key biomarker targets for lung cancer, the properties of GO and Au in biosensing, and the role of AuNP/GO nanocomposites in improving biosensor performance. It further examines the application of electrochemical biosensors for lung cancer biomarker detection, highlighting recent developments. Additionally, the review outlines current challenges limiting clinical translation and PoC implementation, provides recommendations to address these barriers, and discusses future perspectives for improving the detection of low-abundance biomarkers for early lung cancer diagnosis. Ultimately, these technologies seem promising for the development of rapid diagnostic tools equivalent to established platforms such as lateral-flow immunoassays.

## 1. Introduction

**Figure 1:**
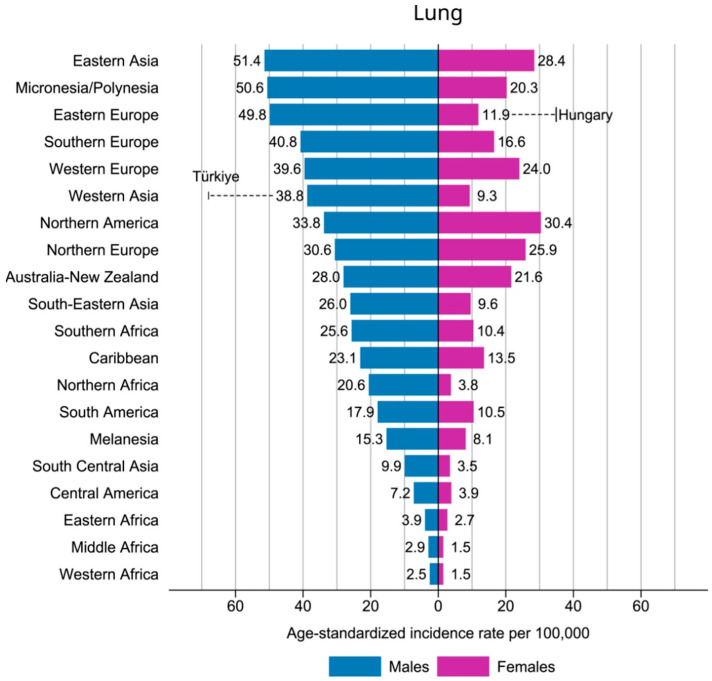
A lung cancer bar chart reporting region-specific incidences, and age-standardized rates by sex for year 2022. Rates are shown by nationality in descending order of the world (W) age-standardized rates in males, whilst the female rates are simultaneously superimposed. Source: GLOBOCAN 2022 [1]. Adapted with permission from [1].

Lung cancer remains the leading cause of cancer-related mortality worldwide, accounting for approximately 25% of all cancer deaths. According to the GLOBOCAN 2022 report, about 2.48 million new cases were diagnosed, including 1.57 million in males and 0.91 million in females, with increasing incidence particularly in low- and middle-income regions [1]. Figure 1 illustrates the regional variations in lung cancer incidence and mortality by sex, reflecting the persistent global burden of the disease.

Despite significant advances in imaging and molecular diagnostic technologies, including artificial intelligence (AI), machine learning, and radiomics [1,2,3], early detection of lung cancer remains challenging. Their clinical implementation is still constrained by limitations in standardization, regulatory validation, and diagnostic reliability. Conventional diagnostic techniques such as chest radiography [4], computed tomography (CT) [4], positron emission tomography (PET) [5], and tissue biopsies [6] remain widely used but are often complicated to operate, costly, and time-consuming. Moreover, these approaches may lack sufficient sensitivity for detecting low-abundance biomarkers associated with early-stage disease, particularly within complex biological matrices.

Electrochemical biosensors have therefore emerged as promising analytical tools capable of detecting trace biomarker concentrations by converting biological recognition events, such as antigen–antibody binding or nucleic acid hybridization, into measurable electrical signals. Recent advances in nanomaterials have further enhanced biosensor performance. In particular, GO and AuNPs have attracted considerable interest due to their complementary electrochemical and surface properties [7]. GO provides a high surface-to-volume ratio and abundant oxygen-containing functional groups that facilitate efficient immobilization of biomolecules such as antibodies, aptamers, and oligonucleotides [8]. AuNPs, in contrast, exhibit excellent electrical conductivity and strong bioconjugation capabilities, enabling signal amplification and improved detection sensitivity [9]. Additionally, both materials demonstrate antifouling properties that help maintain sensor performance in complex biological fluids such as blood, serum, plasma, urine, and saliva, which is critical for PoC diagnostic applications.

This review critically examines recent progress in electrochemical biosensors incorporating GO and AuNPs for lung cancer diagnostics. Particular emphasis is placed on the detection of clinically relevant biomarkers including CEA [10], CYFRA 21-1 [11], NSE [12], and circulating nucleic acids such as circulating tumour (ctDNA) and (microRNA(miRNAs) [13] from minimally invasive biological samples including blood, saliva, and sweat. Despite promising analytical performance, several challenges including surface functionalization, stability, non-specific adsorption, and matrix interference continue to limit clinical translation. By critically analyzing existing literature, this review highlights current advances, remaining gaps, and future prospects for developing robust GO/AuNP-based biosensors for early lung cancer detection.

## 2. Biosensors Fundamentals and Brief History

Biosensors are analytical devices that detect specific analytes by converting biological recognition events into measurable electrical, optical, or thermal signals [14]. The first biosensor was an amperometric glucose electrode developed by Clark and Lyons in 1962 [15], which led to the commercialization of biosensors in 1975. The 1980s marked significant material advancements, including the introduction of immunosensors, followed by the emergence of nanomaterial-enhanced biosensors around 2000. The modern era is characterized by DNA/RNA biosensors, aptasensors, and nanozyme-based platforms that offer enhanced sensitivity. Current trends are shifting toward AI-integrated, smart, multiplexed, and wearable biosensors designed for real-time monitoring with much advancement in miniaturization and multifunctionality [16].

A typical biosensor consists of three main components: a biological recognition element, a transducer, and a signal-processing unit. In biomedical diagnostics, biosensors provide several advantages, including high sensitivity for detecting low-abundance biomarkers and the ability to perform real-time analysis in PoC settings similar to Continuous Glucose Monitoring (CGM) devices. Commercial CGM systems such as the Dexcom G7 CGM System and Abbott FreeStyle employ electrochemical biosensors to monitor glucose levels in interstitial fluid in real time [17]. These systems typically rely on electrochemical methods such as cyclic voltametric, potentiometric, impedimetric, and amperometric methods. Nanomaterials, notably carbon-based such as GO, are incorporated to enhance electrode surface area, biomolecule immobilization, and electron transfer [18]. The addition of AuNPs further improves signal amplification and detection sensitivity, making GO/AuNP nanocomposites widely investigated for biosensor development [19].

In cancer diagnostics, biosensors target biomolecules such as proteins, DNA, RNA, or circulating tumour cells which can indicate disease presence or progression [14]. Importantly, these biomarkers often become dysregulated during early tumorigenesis before structural abnormalities are detectable by imaging techniques such as CT or PET scans. Consequently, developing PoC diagnostic devices capable of rapid and accessible screening, particularly in low- and middle-income countries, is critical, as well as identifying minimally invasive biomarkers for rapid tests.

Electrochemical biosensors are particularly attractive for cancer diagnostics due to their high sensitivity, with detection limits ranging from nanomolar to picomolar concentrations, and in some cases reaching femtomolar or attomolar levels [20,21]. Recent electrode modification strategies using nanocomposites, including GO/AuNP coatings, have further improved signal resolution and selectivity by generating distinguishable electrochemical peaks for individual analytes [22]. These capabilities are particularly relevant for lung cancer diagnostics, where multiplex detection of biomarkers such as CEA, NSE, and CYFRA-21 may improve early detection and disease characterization. The following sections critically examine the role of nanocomposite-based electrochemical biosensors in detecting lung cancer biomarkers, highlighting recent advances, persistent limitations, and future opportunities for clinical translation.

## 3. Graphene Oxide: Properties and Role in Biosensing

**Figure 2:**
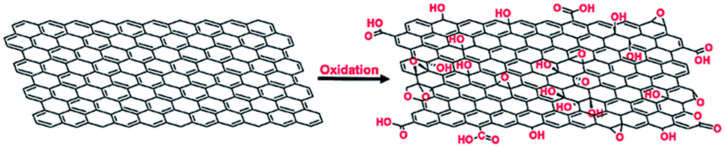
Oxidation of graphene sheet to form graphene oxide [26].

GO is an oxidized derivative of graphene, a two-dimensional (2D) nanomaterial composed of sp^2^-hybridized carbon atoms arranged in a hexagonal lattice, which confers exceptional electrical and thermal conductivity along the basal plane [23]. The atomic configuration and bonding structure of graphene are illustrated in Figure 2. GO is produced through oxidation of graphene, introducing oxygen-containing functional groups such as hydroxyl and epoxy groups on the basal plane and carboxyl and carbonyl groups mainly at the sheet edges [24].

Despite oxidation, the honeycomb lattice is largely preserved with a typical carbon-to-oxygen ratio of 1.5:2.5. These functional groups render GO hydrophilic and chemically reactive, distinguishing it from pristine graphene and enabling versatile surface functionalization for biosensing applications. Consequently, GO has emerged as a widely used nanomaterial in electrochemical biosensors due to its large surface area, favourable electron transfer characteristics, low charge-transfer resistance, and cost-effective synthesis [25].

Multiple synthesis methods exist, including the Brodie [27], Staudenmaier [28], Hoffmann [29], Tour, and Hummers [30] methods, with the latter being most commonly employed. These approaches yield GO with different oxidation levels and functional group distributions, which can significantly influence electrochemical performance and biomolecule immobilization [31]. Permanganate-based methods such as the Hummers and Tour approaches generate higher densities of carbonyl and carboxyl groups, introducing defect sites that enhance catalytic activity and facilitate surface modification with biomolecules or metal nanomaterials [32]. While such defects improve sensing functionality, they also affect conductivity, making synthesis control critical for optimizing biosensor performance.

GO is widely used in electrochemical biosensors for lung cancer detection due to its high surface area and abundant functional groups, which enhance biomolecule immobilization and signal amplification for biomarkers such as CYFRA 21-1, CEA, NSE, (Mucin 1) MUC1, and lung-cancer-associated genetic modifications [33]. Moreover, GO-based nanocomposites also support multiplex detection; for example, GO/AuNP platforms have achieved simultaneous detection of NSE and CEA with pg mL^−1^ sensitivity [34]. These synergistic systems enhance sensitivity and outperform conventional electrodes, highlighting their potential in clinical diagnostics. Furthermore, combining GO with emerging materials such as MXenes [35], conductive polymers [36], Metal Organic Frameworks (MOFs) [37] and greener synthesis approaches is advancing the development of sustainable, portable, and multiplex point-of-care biosensors [38]. Consistent with these findings, Table 1 highlights the improved performance of GO-based biosensors over non-GO electrochemical platforms. It can be noted that the GO-based biosensors outperform non-GO-based biosensors in sensitivity and detection capability while maintaining effective biomarker recognition. The remarkable femtogram-level detection achieved for CYFRA21-1 highlights GO’s potential for ultra-sensitive early cancer diagnosis. The advantages arise from its large surface area, excellent biomolecule immobilization capacity, abundant functional groups, and enhanced signal transduction properties.

## 4. Gold Nanoparticles (AuNPs): Properties and Role in Biosensing

AuNPs are nanoscale gold particles (1–100 nm) composed of crystalline gold atoms and are typically stabilized by ligands to prevent aggregation [43]. They are commonly synthesized through chemical reduction in chloroauric acid (HAuCl_4_), particularly via the Turkevich citrate reduction method which produces spherical AuNPs of ~10–50 nm, while sodium borohydride reduction yields smaller particles; modified citrate methods can produce larger particles [44]. Owing to their high electrical conductivity, optical properties, biocompatibility and large surface area, AuNPs are widely used in electrochemical biosensors for lung cancer biomarker detection. Their colloidal stability facilitates functionalization with biomolecules for selective detection [45]. However, a key limitation is their instability in physiological buffers, where transfer from aqueous synthesis environments often causes aggregation or surface destabilization [46]. Citrate-stabilized AuNPs possess negatively charged surface groups such as carboxylates (COO^−^), hydroxyls (OH^−^) and oxidation products including acetone dicarboxylate (O=C(CH_2_CO_2_ H)_2_), which support functionalization but also increase sensitivity to environmental factors such as pH, ionic strength, temperature and storage conditions [47]. These factors can induce aggregation or dissolution, leading to reduced electron transfer, probe detachment, poor reproducibility and decreased biosensor sensitivity, ultimately limiting device shelf-life, scalability and regulatory approval for PoC applications.

AuNP-facilitated electrochemical biosensors demonstrate exceptional sensitivity for protein biomarker detection, achieving sub-femtomolar limits of detection suitable for early cancer diagnosis [48]. In contrast, non-AuNP biosensors generally detect proteins at higher limits (picomolar–femtomolar), although they can achieve comparable or superior sensitivity for nucleic acids such as miRNAs (0.025 fM) [49]. For example, a non-AuNP biosensor detected lung cancer RNA at 25 attomolar, demonstrating sensitivity comparable to AuNP-based systems [50]. A comparative summary of these platforms is presented in Table 2, highlighting detection performance for protein, DNA, RNA and exosome biomarkers. Overall, AuNP-facilitated biosensors show greater advantages for protein biomarker detection than non-AuNP counterparts. The result suggests that AuNP-based biosensors maintain high specificity while offering superior signal amplification, higher probe loading capacity, enhanced stability, and excellent suitability for detecting nucleic acid and extracellular vesicle biomarkers. These characteristics make AuNPs particularly attractive for the development of highly sensitive electrochemical biosensors for early cancer diagnosis.

AuNPs remain promising nanomaterials for electrochemical biosensing due to their high conductivity, electrocatalytic activity, biocompatibility and ease of functionalization [53,54]. Their surfaces can be modified through covalent or non-covalent interactions with thiols, amines, carboxyl groups, phosphines or silica, enabling integration with other nanomaterials such as graphene oxide to form synergistic sensing platforms [55]. Future research focuses on improving nanoparticle stability, developing hybrid nanostructures and advancing multiplexed, wearable and AI-integrated PoC biosensors for clinical diagnostics [56].

## 5. GO/AuNPs Composites in Enhancing Electrochemical Biosensing of Cancer Biomarkers

GO and AuNPs each exhibit advantageous physicochemical, electronic, and optical characteristics for biosensing applications; however, their integration into GO/AuNP nanocomposites results in markedly improved performance due to synergistic interactions [19]. The hybrid architecture offers an expanded surface area that facilitates dense immobilization of biorecognition elements, thereby enhancing sensitivity, while the increased loading capacity contributes to improved selectivity. Importantly, GO acts as a stabilizing matrix for AuNPs via interfacial interactions, promoting uniform dispersion and limiting nanoparticle aggregation [19]. Concurrently, AuNPs function as conductive nano-bridges between GO sheets and the electrode surface, significantly accelerating electron transfer kinetics and improving overall conductivity. Beyond structural support, GO contributes oxygenated functional groups that enable versatile covalent and non-covalent functionalization strategies, whereas AuNPs provide thiol-reactive surfaces, collectively broadening the range of transduction mechanisms available for biosensing [57]. Nevertheless, the extent of these enhancements is highly dependent on interfacial engineering and composite design, highlighting the importance of controlled synthesis in achieving optimal performance.

**Figure 3:**
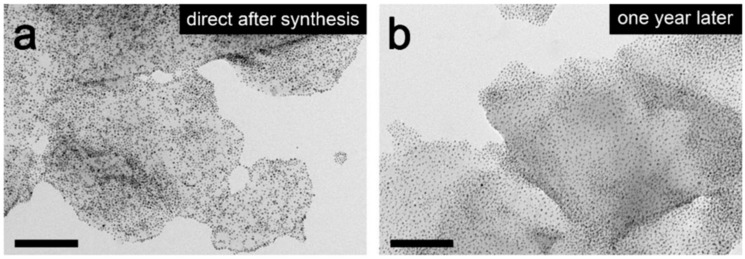
Micrographs of the same sample of CRGO@AuNP hybrid material directly after synthesis (**a**) and a year after (**b**). Scale bars represent length of 200 nm [62].

**Figure 4:**
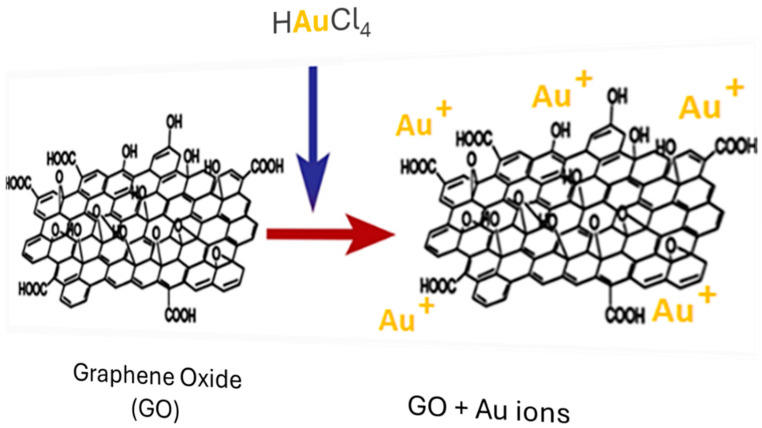
The arrangement of gold ions on the functional groups of GO. Adapted from Al-Ani and coworkers, 2019 [61].

GO/AuNP-based electrochemical biosensors further demonstrate improved stability, biocompatibility, and sensing performance, primarily driven by the strong interfacial synergy between the two components. For instance, functionalised composites such as graphene oxide decorated with AuNP (CRGO@AuNP) exhibit remarkable long-term stability, maintaining structural integrity and preventing nanoparticle aggregation even after one year (Figure 3), in contrast to pristine reduced graphene oxide (rGO) systems that facilitate particle coalescence, thereby emphasizing the role of surface functionalization [58]. Additionally, GO coatings enhance nanoparticle stability in complex biological and environmental matrices while reducing cytotoxic effects, maintaining high dispersion efficiencies (~95%) and suppressing reactive oxygen species (ROS)-induced damage compared to GO alone [59,60]. These stabilizing effects arise from a combination of electrostatic interactions, π–π stacking, and coordination bonding between AuNPs and GO’s oxygen-containing functional groups (Figure 4). However, this stability is not intrinsic and remains highly sensitive to synthesis conditions and surface chemistry [61]. Excessive coordination or improper functionalization may instead induce aggregation, underscoring the need for precise control over composite fabrication.

Beyond structural stability, GO/AuNP hybrids significantly enhance electrocatalytic performance by facilitating rapid electron transfer and increasing catalytic activity. These composites enable highly sensitive detection of analytes, achieving nanomolar detection limits for species such as hydrogen peroxide (H_2_O_2_), outperforming individual GO or AuNP systems. Notably, sensor performance is strongly influenced by the morphology and structural configuration of AuNPs [63]. Despite these advances, variability in interfacial interactions and dependence on material properties present ongoing challenges, limiting reproducibility and large-scale applicability. Consequently, a deeper mechanistic understanding of structure–property relationships is required to design GO/AuNP-based biosensors tailored for specific cancer biomarker detection.

## 6. Biomarker Targets for Lung Cancer

Lung cancer is a malignant lung tumour with various biomarkers that arise from different cell types. The two major types of lung cancer are Non-Small Cell Lung Cancer (NSCLC), which accounts for 85% of cases, and Small Cell Lung Cancer (SCLC), although it constitutes only 10–15% of cases, it is the most aggressive [64]. The five-year survival rate of those inflicted is only 13–15%. NSCLC is further divided into adenocarcinoma, squamous cell carcinoma, and large cell carcinoma, whereas SCLC is subdivided into combined small cell carcinoma and oat cell carcinoma.

The acquisition of appropriate biomarkers is essential in early detection of lung cancer, facilitating disease management, including the administration of prompt and appropriate therapeutic interventions that target the origin of the tumour, reducing progression of the disease and mortality. Blood remains the most promising body fluid for biomarker discovery for early lung cancer detection. Moreover, blood contains a variety of promising biomarkers which include proteins, RNAs, DNAs, as well as the stromal and endothelial cells [65]. Protein biomarkers are measurable in biological samples (e.g., blood, tissue, or urine) with levels or properties capable of indicating specific physiological or pathological states, making them convenient markers of disease presence, progression, and/or response to treatment. Their size, surface chemistry and conformational structures present proteins as suitable targets for electrochemical biosensors, wherein interfacial interactions can be engineered on electrochemical surfaces through specific nanomaterials with enhanced selective capturing, high immobilizing efficiencies, increased electron transfer and robust antifouling capabilities [66]. Diagnostic significance of protein biomarkers is defined by the sensitivity, specificity, selectivity, and limit of detection (LOD). To contextualize the need for advanced sensing platforms, we mainly discuss the most clinically relevant lung cancer biomarkers including, but not limited to CEA, CYFRA 21-1, NSE. The structural conformation and surface chemistry of the protein biomarkers such as size, subunit organization, hydropathy, and charge are important biomarker features that inform the rational design of an optimized electrochemical biosensor as well as the typical concentration ranges and stability in target samples.

### 6.1. Carcinoembryonic Antigen (CEA)

CEA is a 180 kDa acidic glycoprotein, with approximately 50% carbohydrates and comprising several domains. This protein is expressed in the membrane and shed to the extracellular matrix [67,68]. It is mainly produced during fetal development, and because it is involved in the adhesion and modulation processes, tumours with high CEA expression have high metastatic potential [69]. The production of CEA normally ceases at birth; hence, it is not present in detectable amounts in healthy adults. Elevated serum levels of CEA in adults are an indication of some form of cancer, commonly colorectal and lung cancers [70]. The use of CEA as a prognostic and predictive biomarker in lung cancer is still under intensive study, whereas for colorectal cancer, it has been widely validated and used [71]. Tomita et al. [72] evaluated the suitability of CEA as a biomarker in NSCLC. In the study, CEA levels ≥ 5 ng/mL were considered elevated in NSCLC, which is common, and patients presenting with these levels were enrolled, whilst levels above 10 ng/mL were more suggestive of malignancy. These elevated CEA levels stemmed from adenocarcinoma NSCLC subtypes. Evidence of CEA as a predictive marker for early detection, progression or relapse has been validated by several studies [26,67,72,73,74,75], with the conclusion that CEA is an appropriate biomarker for NSCLC. Moreover, these findings underscore the significance of CEA as a biomarker in NSCLC of the adenocarcinoma subtype; further validations are required for other types such as SCLC, and its significance in early detection of lung cancer. To build an optimum functioning biosensor with high sensitivity, specificity and selectivity, the concentration ranges and the physicochemical properties of CEA should be considered, as they play a role in electron transfer and signal amplification. In particular, the large size of CEA should be factored in the design of the biorecognition element, orientation playing an important role in minimizing steric hinderance and increasing CEA binding, thus amplifying the signal.

### 6.2. Cytokeratin 19 (CYFRA 21-1)

CYFRA 21-1 is an intermediate filament protein belonging to the keratin family, with an estimated molecular weight fragment between 18 and 40 kDa [69]. The protein is predominantly α-helical with coiled-coil structures, assuming a rod-like elongated shape that undergoes proteolytic cleavage to produce various molecular weight fragments [76]. Following proteolytic processing, the CYFRA 21-1 epitopes retain their linearity and accessibility, enabling ease of antibody binding. This positions it as a suitable candidate for immunosensors; also, other factors that are important to consider for electrochemical analysis of this protein are its surface charge which is beneficial for tuning and low fouling potential [77]. CYFRA 21-1 is expressed in epithelial cells, and it is a fundamental structural component of the cytoskeleton that maintains cell integrity. CYFRA 21-1 is notably expressed in epithelial derived cancers, such as lung, breast, thyroid, and pancreatic carcinomas. Similar to CEA, CYFRA 21-1 is a circulating biomarker [69], detectable in blood plasma through ELISA tests and electrochemiluminescence (ECLIA) assays as conventional methods of detection [78]. Early detection and monitoring of disease progression remain potential solutions that can reduce costs related to conventional methods of testing. To achieve early diagnosis, the detection limit of the device needs to be low enough to meet the cut-off concentrations of NSCLC. In a study by Nakata et al., 2004, the reported levels of CYFRA 21-1 in the serum of control or health individuals was around 1.2 ng/mL [79], and in another study the levels were found to be 1.96 ng/mL in benign lung disease [80]. It is acceptable that the levels of CYFRA 21-1 that are associated with NSCLC are between 3.0 and 3.3 ng/mL with higher levels indicating advanced stages of the disease and poorer prognosis (>3.5 ng/mL) [40]. A meta-analysis study conducted by Holdenrieder et al. [69] indicates that both CEA and CYFRA 21-1 biomarkers show modest predictive value in post-treatment prognosis of NSCLC, with levels of CYFRA 21-1 displaying better post-treatment prognosis. Therefore, in addition to early detection of NSCLC, CYFRA 21-1 and CEA can be used as predictive tools for monitoring the effectiveness of therapy; moreover, this provides evidence and motivation for designing a multiplex biosensor assay with more robust sensitivity and selectivity.

### 6.3. Neuron-Specific Elanose (NSE)

NSE is a glycolytic enzyme predominantly expressed in neuronal and neuroendocrine tissues and is widely used as a circulating biomarker in lung cancer diagnostics. Elevated NSE levels are strongly associated with small-cell lung cancer (SCLC), an aggressive subtype accounting for approximately 15% of lung cancer cases, where early detection could improve the five-year survival rate from about 17% to nearly 50% [78]. Clinically, NSE is commonly measured using ELISA or chemiluminescent immunoassays alongside imaging methods such as chest X-ray or CT. Satoh et al. reported a diagnostic cut-off of 14.5 ng/mL in a cohort of 417 lung cancer patients, with the 95th percentile for NSCLC at 20.5 ng/mL and values above 70 ng/mL frequently observed in SCLC [78]. However, NSE lacks strict disease specificity, as elevated levels are also reported in NSCLC with neuroendocrine differentiation, carcinoids, neuroblastomas, and several non-malignant neuronal conditions [81,82]. This biomarker overlap reduces the reliability of single-analyte diagnostics and has motivated the development of multiplex detection strategies [83]. Conventional rapid tests such as lateral flow assays often lack the sensitivity required to detect early-stage NSE concentrations [84], while laboratory chemiluminescence assays offer higher sensitivity but remain unsuitable for decentralized testing. Additionally, hemolysis presents a major analytical challenge because erythrocyte rupture releases NSE, resulting in falsely elevated readings [85,86]. Despite these limitations, NSE has been detected in multiple biofluids including serum, urine, saliva, and sweat [87,88,89], highlighting its potential for minimally invasive diagnostics. These challenges underscore the need for highly sensitive and selective point-of-care platforms. Nanomaterial-enhanced electrochemical biosensors offer a promising approach for improving NSE detection when sensor architectures are optimized for efficient biomarker recognition.

### 6.4. MicroRNAs (miRNA)

MiRNAs are increasingly recognized as prevailing diagnostic biomarkers for lung cancer due to their small size, remarkable stability in biofluids (e.g., serum, plasma, sputum, and exhaled breath condensate), and their regulatory role in oncogenic pathways such as cell proliferation, apoptosis, angiogenesis, and metastasis [90]. Specific miRNAs, including miR-21 [91,92], miR-155 [93], miR-210 [94], and miR-126 [95], have been consistently reported to exhibit differential expression profiles between lung cancer patients and healthy or benign disease cohorts, enabling early-stage detection through minimally invasive approaches. Their resistance to enzymatic degradation and presence within extracellular vesicles further enhance their suitability for biosensing applications [96]. However, despite these advantages, several critical challenges limit their translation into reliable diagnostic biosensors. Firstly, there are challenges in pre-analytical and analytical variability, such as differences in sample collection, RNA extraction efficiency, and normalization strategies. Secondly, the expression of certain miRNAs is not entirely cancer-specific, as overlaps exist with inflammatory and other non-malignant conditions, thereby reducing diagnostic specificity. Thirdly, miRNAs are often present at ultra-low concentrations, necessitating signal amplification strategies or highly sensitive nanomaterial-based transducers, which can increase system complexity and cost. Furthermore, issues related to probe design, non-specific binding, and surface fouling in complex biological matrices remain significant barriers. Collectively, these limitations underscore a key research gap in the development of miRNA-based biosensors, where future efforts should prioritize multiplexed detection platforms, robust surface functionalisation strategies, and integrated amplification-free sensing systems to achieve clinically relevant sensitivity, specificity, and reproducibility [97].

GO/AuNPs nanocomposites have been extensively employed for electrochemical detection of cancer biomarkers including PSA, CEA, HER2, MUC1, and miRNAs such as miR-21 and miR-155, achieving LODs from pg/mL to aM levels due to the synergistic effect of GO’s high surface area for probe loading and AuNPs’ excellent conductivity and biocompatibility.

## 7. Conventional Methods of Diagnosing Lung Cancer

Conventional diagnostic techniques for lung cancer biomarker detection, including enzyme-linked immunosorbent assays (ELISA) [98], chemiluminescence immunoassays (CLIA) [99], and polymerase chain reaction-based methods [100], remain the gold standard in clinical diagnostics due to their established reliability, high specificity, and widespread clinical acceptance. However, these methods often require sophisticated instrumentation, trained personnel, centralized laboratory facilities, and relatively long assay times, which may limit their application in point-of-care settings.

Table 3 shows comparitive studies of conventional diagnostic methods and GO/AuNP-based electrochemical biosensors, which demonstrates the significant analytical advantages offered by nanomaterials. For example, Izawa et al. [101] showed that a conventional chemiluminescence immunoassay detects CEA and CYFRA21-1 in the ng/mL range, whereas graphene-based electrochemical biosensors have achieved detection limits of 0.148 pg/mL and 0.04 pg/mL [102], respectively. Similarly, AuNP-assisted platforms have enabled CYFRA21-1 detection at 0.3 pg/mL [23]. These improvements can be attributed to the large surface area and abundant functional groups of graphene oxide, which facilitate high-density biomolecule immobilization, together with the excellent electrical conductivity and signal amplification properties of AuNPs. Consequently, GO/AuNP composites offer substantially improved sensitivity for protein, nucleic acid, and extracellular vesicle biomarkers compared with conventional diagnostic approaches, while maintaining the potential for rapid and point-of-care testing. However, issues related to reproducibility, biofouling, long-term stability, and large-scale clinical validation remain significant challenges for their translation into routine clinical practice.

## 8. Electrochemical Biosensor Systems for the Detection of Lung Cancer Biomarkers

### 8.1. Electrochemical Immunosensor

Electrochemical immunosensors rely on specific antibody–antigen binding events to transduce biomarker recognition into measurable electrical signals, offering a platform for lung cancer diagnostics [103]. Clinically relevant targets such as CEA, NSE and CYFRA21-1 have been successfully detected using nanostructured interface-based immunosensors. For example, a gold particle deposited on a molybdenum disulphide (MoS_2_@Au) nanozyme-based platform achieved a detection limit of 0.1 pg/mL in serum, while a more complex system incorporating AuNPs@MoS_2_/rGO as a capture layer and silver nanoparticles coated onto cobalt ferrite (CoFe_2_O_4_@Ag-labelled) antibodies reached an even lower detection limit of 3 fg/mL [104]. These improvements stem from the specificity of an antibody to its antigen together with an increased surface area and accelerated electron transfer. However, the reliance on multiple nanomaterials raises concerns regarding fabrication cost, batch-to-batch consistency and long-term stability. This highlights a trade-off between achieving ultra-high sensitivity and maintaining practical manufacturability, suggesting that future designs should prioritize simplified architectures without compromising performance. Similarly, Linlin et al. [103] developed the snowflake Cu_2_S/Pd/CuO nanocomposite to construct an original label-free electrochemical immunosensor for the ultrasensitive detection of CEA levels. The immunosensor successfully detected CEA in human serum, and the results are consistent with those of the commercial electrochemical immunosensor. This convergence in performance underscores the importance of stable antibody attachment and favourable orientation, where materials such as hydroxyapatite and chitosan enhance biocompatibility, and 3-aminopropyl, triethoxysilane (APTES) functionalization promotes covalent binding that preserves antigen accessibility. However, translation beyond laboratory conditions remains limited.

Overall, electrochemical immunosensors show strong potential for early lung cancer detection, particularly when incorporating AuNPs and graphene-based materials. However, while current systems achieve excellent sensitivity and selectivity, challenges related to reproducibility, scalability and clinical translation persist. Addressing these limitations through controlled material design and simplified fabrication strategies will be important for advancing GO/AuNP-based platforms toward practical diagnostic applications.

### 8.2. Electrochemical Aptasensors

Aptamers, derived from the Latin word “aptus” meaning “to fit,” are synthetic single-stranded DNA or RNA oligonucleotides selected from artificial nucleic acid libraries for their high specificity and affinity toward diverse molecular targets [105]. Often described as “chemical antibodies,” aptamers recognize targets through their unique three-dimensional folded structures, enabling their integration into biosensors known as aptasensors. In electrochemical aptasensors, the aptamer–target interaction is transduced into measurable electrical signals. The incorporation of conductive nanomaterials such as carbon nanotube GO and gold AuNPs significantly enhances signal transduction. As discussed previously, the synergistic interaction between AuNPs and GO improves electron transfer and sensing performance. Thiolated aptamers readily form stable self-assembled monolayers on AuNP surfaces through Au-S bonding, promoting an optimal orientation for target binding and increasing probe loading. Meanwhile, GO contributes a large two-dimensional surface area and abundant functional groups (carboxyl, hydroxyl, and epoxy), providing additional immobilization sites and improving biosensor sensitivity [105].

Several AuNP-based aptasensors, either alone or combined with graphene/GO, have demonstrated remarkable analytical performance for cancer biomarker detection in both buffer systems and complex biological matrices, as summarized in Table 4. These systems frequently achieve detection limits in the pico- to femtomolar range, indicating strong analytical sensitivity even in serum samples. For example, a multiplex CEA/NSE aptasensor achieved an improved LOD of 2 pg/mL and demonstrated clinical applicability when validated using serum samples [106]. This platform utilized amine-functionalized graphene-thionine-AuNPs and Prussian blue-poly-3,4-ethylenedioxythiophene (PB-PEDOT)-AuNPs to modify the electrode and immobilize antibodies, thereby enhancing electrochemical signal amplification.

It is important to note that the ultralow limit of detection achieved in this work does not arise solely from the nanocomposite. The high sensitivity results from a synergistic effect between the aptamer and the nanomaterial. The aptamer provides high binding affinity and specificity toward the target analyte, enabling efficient capture at fM–pM concentrations, while the nanocomposite significantly amplifies the electrochemical signal through enhanced electron transfer and catalytic activity. Control experiments omitting either the aptamer or the nanocomposite led to LODs that were 2–3 orders of magnitude higher, confirming that both molecular recognition and signal amplification are essential for the observed performance [107].

**Table 4:** Performance examples of AuNPs-based aptasensors with or without carbon-based nanomaterial.

Biomarker	Sensor Type	Nanomaterial	Analytical Performance	Biological Validation	Reference
CEA	Label-free EIS aptasensor	AuNP electrode	LOD ~2.4 pg/mL (buffer); ~3.8 pg/mL	Clinical human serum	[108]
CEA	Sandwich-type electrochemical aptasensor	Hemin-rGO-AuNPs + HRP-nanoflowers	LOD ~29 fg/mL (100 fg/mL–100 ng/mL linear range)	Clinical human serum	[109]
CEA	Electrochemical aptasensor	NCMTs@Fe_3_O_4_@Cusilicate/ConA was used to enrich CEA, Au nanoclusters were used as the probe in the sensor	5.38 pg/mL (Linear range 0.03 to 6 ng/mL)	Clinical human serum	[108]
CEA & NSE (multiplex)	Microfluidic paper aptasensor	Amine-functionalized graphene-thionine-AuNPs + PB-PEDOT-AuNPs	CEA LOD ~2 pg/mL; NSE LOD ~10 pg/mL (Linear range 0.01–500 ng/mL & 0.05–500 ng/mL)	Clinical human serum	[106]

HRP—Horseradish peroxidase; NCMTs@—Nitrogen-doped carbon microtubes.

Overall, these studies demonstrate the strong analytical capabilities of GO/AuNP-based aptasensors; however, most reported systems remain largely validated in controlled laboratory conditions, highlighting the need for further studies focusing on reproducibility, long-term stability, and large-scale clinical validation for practical diagnostic applications.

### 8.3. DNA/RNA-Based Electrochemical Sensors

DNA/RNA-based electrochemical biosensors, commonly named Genosensors, exploit nucleic acid hybridisation for the highly specific detection of genetic biomarkers [110]. Typically employing ssDNA, RNA, or PNA probes, these systems enable identification of complementary DNA/RNA sequences, including miRNAs, mutations, and epigenetic markers [111]. While ssDNA remains the preferred probe due to its stability and ease of functionalisation [112], overall sensor performance depends strongly on probe immobilization efficiency and hybridisation kinetics. Although nucleic acids provide excellent molecular recognition [113], signal transduction often requires amplification strategies or nanomaterial integration, indicating that intrinsic specificity alone is insufficient for achieving clinically relevant sensitivity. Moreover, the versatility of DNA as a structural and signalling element enables indirect detection of non-nucleic acid targets [114], but this added complexity may compromise reproducibility and increase assay variability. Among nucleic acid biomarkers, microRNAs (miRNAs) have emerged as key targets for electrochemical biosensor development in lung cancer diagnostics, particularly in DNA-based sensing platforms.

MiRNAs are among the most prominent targets for genosensor development in lung cancer diagnostics [115], with miRNA-21 and related panels widely investigated [116,117,118]. Nanocomposite-modified electrodes, particularly those incorporating AuNPs with GO, rGO or GQDs, have driven significant improvements in detection limits from picomolar to femtomolar levels as shown in Table 4. However, these gains are largely material-driven rather than probe-driven, underscoring the dependence of genosensor performance on electrode engineering. Additionally, most reported platforms are validated in buffer or spiked samples, limiting confidence in their clinical applicability and highlighting the need for robust validation in heterogeneous biological environments.

Multiplex genosensors further enhance diagnostic potential by enabling simultaneous detection of multiple miRNAs. For example, AuNPs/GQDs/GO-based platforms achieved femtomolar detection of miRNA-21, miRNA-155 and miRNA-210 with distinct voltammetric signals [119], while multi-miRNA panels combined with LDCT data demonstrated high predictive accuracy (AUC = 0.989) for lung cancer [120]. Despite these advances, multiplexing introduces challenges such as signal cross-reactivity, probe interference and increased fabrication complexity. Consequently, while DNA/RNA-based electrochemical biosensors show strong promise, their clinical translation remains constrained by reliance on complex nanomaterials, limited real-sample validation, and challenges in achieving reproducible, scalable designs. Comparisons of LODs of different miRNA biomarkers on different nanocomposites are shown in Table 5.

### 8.4. Electrochemical Nanozyme-Based Biosensors

**Figure 5:**
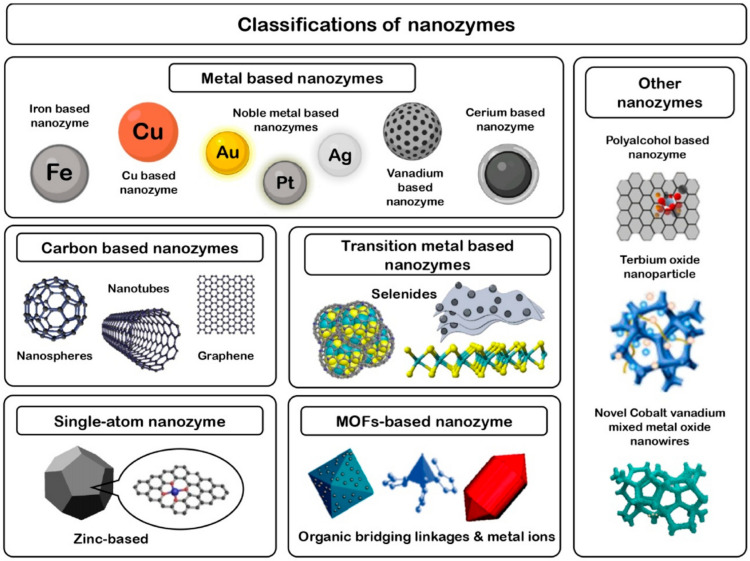
Classification of nanozymes and examples [130]. Adapted with permission from [130].

Nanozyme-based biosensors, as shown in Figure 5, utilize nanomaterials with enzyme-like catalytic properties to enable biomarker detection, offering a compromise between the high activity of natural enzymes and the robustness of synthetic materials [127,128]. While their stability, tunability and cost-effectiveness have driven increasing interest in electrochemical sensing platforms, their catalytic behaviour is fundamentally governed by surface properties, making performance highly dependent on surface engineering [129]. Although noble metals such as AuNP, metal oxides and carbon-based nanozymes such as GO dominate current designs, issues such as aggregation, ligand interference and susceptibility to matrix effects (e.g., salts and small molecules) limit reliability in complex biological samples [130,131]. Thus, despite their advantages, nanozymes often require careful functionalization to balance catalytic efficiency with selectivity and antifouling capability.

Hybrid nanozyme systems are frequently adopted to address these limitations. For example, GO/AuNP composites enhance catalytic activity and stability compared to GO alone, owing to synergistic interactions that promote electron transfer and facilitate H_2_O_2_ reduction [132,133,134]. Ligand modification further tunes catalytic sites and improves electron transfer pathways, but excessive surface modification may alter active sites and reduce reproducibility. This highlights a key limitation: while hybridisation improves performance, it also increases system complexity and sensitivity to synthesis conditions.

Despite their potential, nanozyme-based electrochemical biosensors remain underexplored for clinically relevant lung cancer biomarkers such as CEA, CYFRA 21-1, NSE and miRNAs. Existing studies demonstrate promising sensitivity; for instance, a Pd/Pt nanozyme sensor achieved an LOD of 0.12 pg/mL for CEA with good agreement with clinical assays [135]. However, many reported systems, including AuNP/GO-based platforms (Table 6), are validated primarily in buffer or simplified models and often exhibit limited sensitivity at clinically relevant concentrations, indicating a gap between analytical performance and real-world applicability.

**Figure 6:**
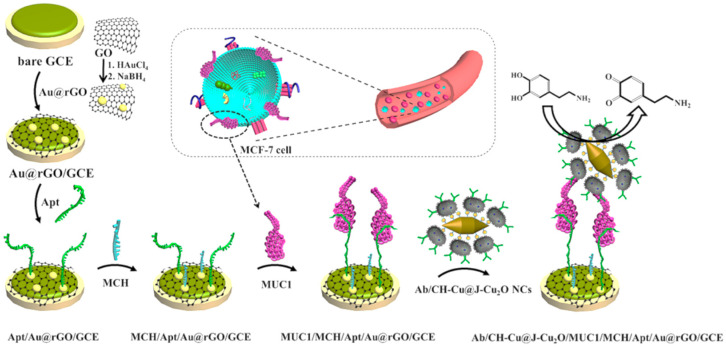
Schematic diagram of nanozyme-enhanced electrochemical protein detection fabrication process of the sandwich-type electrochemical MUC1 sensor [140]. Adapted with permission from [140], Applied nanomaterials.

Recent designs incorporating multifunctional nanozymes, such as CH-Cu@J-Cu_2_O for MUC1 detection, demonstrate improved sensitivity (LOD 0.085 pg/mL) through cascade catalytic mechanisms (Figure 6) [140]. While such architectures highlight the versatility of nanozymes, they also introduce additional design complexity and potential reproducibility challenges. Overall, nanozyme-based electrochemical biosensors offer stable and adaptable platforms suitable for harsh conditions, but their limited intrinsic specificity and potential toxicity remain critical barriers. Integrating nanozymes with biological recognition elements (e.g., aptamers or antibodies) is therefore essential to enhance selectivity and enable practical clinical translation.

## 9. Challenges and Considerations

As much as GO/AuNP-based electrochemical biosensors show promising opportunity in POC diagnostics, there are few challenges that exist which limit their practicality in clinical practise such as selectivity, reproducibility, scalability and issues with fouling.

Selectivity: In complex biological matrices (e.g., serum), non-specific binding can interfere with accuracy. Surface functionalization must be optimized to ensure specificity. A few strategies can be exploited, such as controlled orientation methods for immobilizing antibodies and using biorecognition that bind the target with high affinity and specificity such as nucleic acid-based ones, aptasensors and DNA/RNA-based biosensors [141]. Additionally, specificity can be further enhanced with the use of molecularly imprinted polymers (MIPs), which can be applied as an extra layer of coat, acting as a binding receptor for the target [142].

Reproducibility and Stability: Variability in GO/AuNPs synthesis (e.g., particle size, dispersion) can affect sensor performance, necessitating standardized protocols [141]. This can be improved with the use of molecules that act as templates for synthesis and/or as enablers of nucleation and growth of the nanomaterial. Ionic liquids are green neoteric solvents that have been recently used for the synthesis of nanomaterials, supporting nucleation and growth, and serving as templates and stabilizers [143]. Stability is crucial in developing a robust biosensing system, ensuring reproducibility, longer shelf life, and preserving electrochemical sensing properties such as electron conductivity, biorecognition and catalytic activity. Maintaining colloidal stability and preventing aggregation of nanoparticles is important for ensuring prolonged and consistent analytical performance. Various approaches have been employed to enhance the stability of nanoparticles, including surface engineering with polyethylene glycol (PEG), zwitterionic polymers, covalent immobilization, the use of core–shell nanostructures and capping with agents such as trisodium citrate and sodium dodecyl sulphate (SDS). New trends have also emerged, including the use of green materials, as mentioned, ionic liquids and green deep eutectic solvents, and bio-inspired and biomimetic agents such as polydopamine (PDA), peptides, proteins and cell membrane proteins [144].

Clinical Translation: While promising in research, these sensors require validation with large patient cohorts and regulatory approval for clinical use. Only preclinical validations have been done to demonstrate proof-of-concept, including the spiking of biological samples such as serum and urine, and testing of patient serum samples. Biofouling presents a major obstacle when complex biological samples are tested; hence, it is inherently linked to biosensor underperformance in clinical settings. Although nanomaterials have improved the antifouling properties of biosensors, challenges remain. Greater improvements have been achieved through hybrid use—wherein the large surface area of nanoparticles is coated to prevent nonspecific adsorption of biomolecules. The most applied antifouling coat materials include PEG, hydrogels and zwitterionic polymers [145]. Next-generation antifouling strategies have been reviewed by Dhaffouli [146], highlighting emerging methods in green synthesis, biomimetic coatings, and multifunctional hybrid materials. This includes the design of SMART material which is stimuli-responsive and adaptive whilst being able to self-clean. Since the mechanisms of biofouling are poorly understood, computational methods can be employed to gain more insight and consequently build predictive and optimization algorithms.

Regulatory approval: The standardization of GO/AuNP protocols is not yet well-established, including health and safety concerns of GO and AuNP exposure. Although the risks may be minimum due to low concentrations of GO and AuNPs, the protocols still need to be proved to be safe. Waste management of the devices may also be a health risk concern. This serves as an opportunity for integrating green material into developing the devices. Ionic solvents are well-suited, which can be used for waste valorisation, and they can be employed for metal and nanoparticle recovery through their tuneability. This could be a strategic feed into sustainability and recyclability, aligning with principles of circular economy [143].

Economic feasibility: GO/AuNP-based electrochemical biosensors offer a cost-effective alternative to conventional techniques such as ELISA, chemiluminescence assays, and PCR due to their simpler instrumentation and rapid analysis. However, the production of high-quality nanomaterials, surface functionalization, and sensor fabrication can increase the costs. Therefore, the development of affordable and scalable fabrication strategies remains crucial for their widespread adoption in point-of-care diagnostics [146].

Manufacturability: The large-scale manufacturing of GO/AuNP-based electrochemical biosensors remains a challenge due to difficulties in achieving consistent nanomaterial synthesis and sensor fabrication. Variations in nanomaterial properties can affect sensor performance, while scalable production requires standardized fabrication and quality control processes. Although techniques such as screen-printing and roll-to-roll manufacturing show promise, further optimization is needed to support commercial and clinical deployment [25].

Table 7 summarizes the main lung cancer biomarkers, sensing strategies, analytical performance, advantages, and limitations of GO/AuNP-based platforms.

## 10. Future Perspectives of GO/AuNPs Electrochemical Biosensors

As current research and technology progresses, electrochemical biosensors are most likely to evolve from laboratory prototypes to clinically viable tools, offering improved sensitivity, portability, and integration into broader diagnostic ecosystems. The future of GO/AuNP-based electrochemical biosensors for lung cancer detection holds immense potential, expedited by advances in nanobiotechnology, bioinformatics, artificial intelligence, and personalized medicine. Below are key future perspectives and directions for their development and application in lung cancer detection.

1. Enhanced sensitivity and specificity—Future conjugates of GO/AuNPs may include additional nanomaterials (metal–organic frameworks of single-atom catalysts) to further enhance electron transfer and signal capabilities. This will further enhance the LOD, thereby enabling ultra-early detection of lung cancer. Advances in surface chemistry will allow precise and accurate configuration of GO/AuNPs with aptamers, nanobodies, or CRISPR-Cas systems to enhance specificity for lung cancer-specific biomarkers (e.g., mutated EGFR, miR-155, or novel exosomal markers). This could reduce false positives in complex biological samples like blood or sputum [121].
2. Multiplexed biosensors which enable simultaneous detection of biomarkers (e.g., proteins, miRNAs, and VOCs) on a single GO/AuNP platform, providing a comprehensive diagnostic profile for lung cancer subtypes and stages which can be cost-effective and less laborious by cutting out the running of multiple assays [147]. Moreover, multiplexing enhances sensitivity with minimum sample volume requirements. Miniaturization and Point-of-Care (POC) Applications.
3. Wearable and Portable Devices: GO/AuNP biosensors could be integrated into microfluidic chips or wearable devices for real-time monitoring of lung cancer biomarkers in breath, saliva, or sweat. This would enable non-invasive, at-home screening, particularly for high-risk populations (e.g., smokers, industrial workers). Real-time monitoring offers the advantage of prescribing tailor-made regimens which aligns with precision medicine [148].
4. Smartphone Integration: Pairing these biosensors with smartphone-based electrochemical readers could enable access to lung cancer diagnostics, especially in low-resource settings—provided that the necessary resources from state support systems are in place. GO/AuNPs’ stability and low-cost fabrication make them ideal candidates for such scalable technologies. Integration of Smartphones can facilitate data storage and exchange for health care systems, which enables monitoring patient progress, and data/epidemiological analysis for research purposes [149].
5. Lab-on-a-Chip Systems: Combining GO/AuNPs with lab-on-a-chip platforms could streamline sample processing, biomarker detection, and data analysis in a single, compact device, reducing turnaround time from hours to minutes [150].
6. Integration with Artificial Intelligence (AI) and Big Data AI-Driven Diagnostics: Machine learning algorithms could analyze electrochemical signals from GO/AuNP biosensors to identify patterns associated with lung cancer detection and progression, improving diagnostic accuracy beyond human interpretation. This could also help differentiate benign conditions (e.g., inflammation) from malignancy. Additionally, learned patterns can help differentiate analyte response from background noise, improving selectivity and sensitivity [148].
7. Biomarker Discovery: By coupling GO/AuNP sensors with AI, researchers could identify novel lung cancer biomarkers from large datasets (e.g., proteomic, or genomic profiles), expanding the range of detectable targets and enhancing early detection capabilities [151].
8. Personalized Medicine and Theragnostics (therapeutics and diagnostics). Patient-Specific Sensors: GO/AuNPs could be customized to detect individual biomarker signatures, reflecting the genetic and molecular heterogeneity of lung cancer (e.g., NSCLC vs. SCLC). This aligns with the shift toward personalized diagnostics and treatment plans [152].

## 11. Conclusions

The future of GO/AuNP electrochemical biosensors in lung cancer detection lies in their evolution into sensitive, portable, and intelligent diagnostic tools. By addressing current challenges (e.g., selectivity, scalability) and leveraging emerging technologies (e.g., AI, microfluidics), these biosensors could transform early detection, improve patient outcomes, and reduce the mortality associated with lung cancer. Within the next decade, we may see GO/AuNP-based platforms integrated into routine healthcare, offering a powerful weapon in the fight against one of the deadliest cancers worldwide. Sustained interdisciplinary collaboration spanning materials science, oncology, and data science will be key to realizing this potential of developing multiplexed electrochemical biosensors that meet early detection capabilities in cancer disease management.

## Figures and Tables

**Table 1:** Comparison of GO-based and non-GO-based electrochemical biosensors for lung cancer detection.

Biosensor Type	Biomarker	Detection Method	Layer Content	LOD	Linear Range	Reference
GO-facilitated	CEA	Electrochemical (GO-modified electrode)	GO	0.1 ng/mL	0.1–100 ng/mL	[39]
	PSA	Electrochemical (GO-modified electrode)	GO/AuNPs/aptamer/GCE	0.17 pg/mL	Not specified	[40]
	PSA	Electrochemical (GO-modified electrode)	GO/AuNPs/aptamers/GCE	3.4 pg/mL	Not specified	[41]
	CYFRA21-1	Surface Plasmon Resonance (SPR)	Carboxyl-GO enhanced	1 fg/mL	Not specified	[41]
Non-GO-facilitated	CEA	Electrochemical (carbon nanotubes)	Carbon nanotubes	0.5 ng/mL	Not specified	[40]
	CYFRA21-1	Optical (aper-based microfluidics)	Gold nanoparticles	0.1 ng/mL	Not specified	[42]

**Table 2:** Examples of AuNP-based and non-AuNP-based electrochemical biosensors.

Biosensor Type	Biomarker Type	Detection Method	Layer Content	Detection Limit	Specificity	Ref.
AuNP-facilitated	DNA	GO/AuNP electrochemical	GO/AuNP/aptamer/GCE	2.14 fM (CYFRA 21-1)	High	[51]
	Proteins	GO/AuNP electrochemical	GO/Au/aptamer/GCE	Not specified	High	[42]
Non-AuNP-facilitated	Protein	Electrochemical (GO-based)	GO/GCE	0.23 fM (IL-6)	High	[49]
	Protein	Electrochemical GO-based	GO/GCE	~5.6 pM (CEA, hypothetical)	High	[52]
	DNA	Electrochemical (GO-based)	GO/GCE	pM-fM range	High	[50]

**Table 3:** Comparitive studies of conventional diagnostic methods and GO/AuNP-based electrochemical biosensors.

Biomarker	Conventional Method	LOD/Sensitivity	Ref.	GO/AuNP-Based Electrochemical Biosensor	LOD/Sensitivity	Ref.
CEA	chemiluminescence immunoassay	0.3 ng/mL	[101]	(rGO)-based biosensor for CEA	0.148 pg/mL	[102]
CYFRA21-1	chemiluminescence immunoassay	0.1 ng/mL	[101]	rGO-based biosensor for CYFRA21-1	0.04 pg/mL	[23]
CYFRA21-1	ELISA	3.3 ng/mL	[99]	AuNP-amplified dual-mode biosensor	0.3 pg/mL	[23]

**Table 5:** DNA-based biosensors modified with various nanomaterials for detection of different miRNA biomarkers.

Target	Nanocomposite/Materials	AuNPs	GO/rGO/GQDs	LOD	Sample(s) Tested	Reference
miRNA-21 & miRNA-155	AuNPs/rGO paper	✔️	rGO	miR-21: 12.0 nM; miR-155: 25.7 nM	Buffer; spiked human serum	[121]
miRNA-21	TH/rGO/CMK-3/AuNPs	✔️	rGO	0.046 fM	Buffer; spiked human serum	[119]
miRNA-21	Graphene/AuNPs/polypyrrole	✔️	Graphene	0.020 fM	Buffer; spiked serum	[122]
miRNA-21, miRNA-155, miRNA-210	AuNPs/GQDs/GO film	✔️	GO + GQDs	miRNA-21: 0.04 fM; miRNA-155: 0.33 fM; miRNA-210: 0.28 fM	Buffer; spiked serum	[123]
miRNA-486-5p	Fe-embedded carbon nanotubes (electrocatalytic)	✖️	✖️	~0.85 fM	Lung cancer cell extracts	[124]
miRNA-541	GQDs	✖️	✔️	0.7 fM	Buffer; spiked in diluted human plasma	[125]
miRNA-122	Label-Free genosensor	✖️	✖️	0.1 pmol	Buffer	[126]

1. TH—thionine; CMK—Carbon Mesostructured by KAIST (Korean University); 2. GQD—graphene quantum dots.

**Table 6:** AuNP/GO hybrids with peroxidase-like activity in biosensor systems developed for detection of cancer.

Nanozyme Composition	Nanozyme Activity	Detection Mode	Linear Range	LOD	Samples Tested	Reference
Au@PtNP/GO	Peroxidase-like	Electrochemical (TMB oxidation signal)	1 μM–3 mM	0.2 μM	Buffer	[136]
Au-PEI/GO (AuNPs on GO)	Peroxidase-like	Amperometric	0.5–1680 μM	0.2 μM	Buffer	[137]
GN/AuNPs (graphene + AuNPs)	Peroxidase-like	Amperometric	0.5–500 μM	0.22 μM	Cell line	[138]
RGO-Au-PTBO (reduced GO + Au)	Peroxidase-like	Amperometric	5–1077.1 μM	0.2 μM	Cell line	[139]
GO@Au NCs (folic acid-conjugated)	Peroxidase-like	Colorimetric (TMB-H_2_O_2_)	Qualitative analysis	Qualitative analysis	Cell line	[140]

**Table 7:** A summary of lung cancer biomarker sensing strategies, performance, advantages and limitations.

Biomarker	Sensing Platform	Analytical Performance (Range/LOD)	Advantages	Limitations	Reference
CEA	GO/AuNP or GO-AuNP hybrids on GCE/SPE immunosensor	Range 0.01–500 ng/mL LOD 2–100 fg/mL	High surface area/plasmonic/catalytic synergy. Excellent signal amplification. Improved biocompatibility.	Potential aggregation of NPs; GO insulating (often needs reduction); matrix interference in complex biofluids	[135]
CYFRA 21-1	rGO-based or carboxyl-GO SPR/electrochemical; AuNP hybrids	Range 0.05–1000 ng/mL LOD 0.01 ng/mL to 5 pg/mL	Strong functionalization via oxygen groups. Ultrasensitive for NSCLC. Label-free options possible.	Specificity challenges in some elevated conditions	[137]
NSE	AuNP-GO nanocomposite (chitosan-mediated). 3D GO/AuNP hybrids on GCE/SPE	Range 0.1–2000 ng/mL. LOD 0.05 ng/mL to 3 pg/mL. Green synthesis possible	Fast electron transfer. Wide dynamic range for SCLC monitoring.	Potential fouling in serum	[138]
Multi-biomarker (CEA + NSE/AFP)	GO/AuNP or GO/AuNP multiplexed platforms	Range Sub pg/mL to ng/mL. LOD 2–10 pg/mL	Simultaneous detection improves accuracy/specificity; synergistic GO + AuNP effects (higher EASA, better ET).	Increased complexity in fabrication and data interpretation. Cross-reactivity risks	[139,140]

## Data Availability

This is a review article; there is no available data.

## References

[1] Fu M., Peng Z., Wu M., Lv D., Lyu S., Li Y. (2025). Assessing the African Burden of Breast Cancer: A Demographic Analysis Using Global Cancer Observatory 2022. Eur. J. Surg. Oncol..

[2] Tantray J., Patel A., Parveen H., Prajapati B., Prajapati J. (2025). Nanotechnology-Based Biomedical Devices in the Cancer Diagnostics and Therapy. Med. Oncol..

[3] Singh S., Hasan M.R., Sharma P., Narang J. (2022). Graphene Nanomaterials: The Wondering Material from Synthesis to Applications. Sens. Int..

[4] Kim J., Kim K.H. (2020). Role of Chest Radiographs in Early Lung Cancer Detection. Transl. Lung Cancer Res..

[5] Gallamini A., Zwarthoed C., Borra A. (2014). Positron Emission Tomography (PET) in Oncology. Cancers.

[6] Hofman P. (2019). The Challenges of Evaluating Predictive Biomarkers Using Small Biopsy Tissue Samples and Liquid Biopsies from Non-Small Cell Lung Cancer Patients. J. Thorac. Dis..

[7] Lazar O., Marinoiu A., Raceanu M., Pantazi A., Mihai G., Varlam M., Enachescu M. (2020). Reduced Graphene Oxide Decorated with Dispersed Gold Nanoparticles: Preparation, Characterization and Electrochemical Evaluation for Oxygen Reduction Reaction. Energies.

[8] Chen D., Feng H., Li J. (2012). Graphene Oxide: Preparation, Functionalization, and Electrochemical Applications. Chem. Rev..

[9] Liu L., Xia N., Liu H., Kang X., Liu X., Xue C., He X. (2014). Highly Sensitive and Label-Free Electrochemical Detection of MicroRNAs Based on Triple Signal Amplification of Multifunctional Gold Nanoparticles, Enzymes and Redox-Cycling Reaction. Biosens. Bioelectron..

[10] Khonyoung S., Mangkronkaew P., Klayprasert P., Puangpila C., Palanisami M., Arivazhagan M., Jakmunee J. (2024). Point-of-Care Detection of Carcinoembryonic Antigen (CEA) Using a Smartphone-Based, Label-Free Electrochemical Immunosensor with Multilayer CuONPs/CNTs/GO on a Disposable Screen-Printed Electrode. Biosensors.

[11] Aydn E.B., Aydn M., Sezgintürk M.K. (2023). Novel Electrochemical Biosensing Platform Based on Conductive for Sensitive and Selective Detection of CYFRA 21-1. Sens. Actuators B Chem..

[12] Yu X., Li X., Zhang S., Jia Y., Xu Z., Li X., Chen Z., Li Y. (2021). Ultrasensitive Electrochemical Detection of Neuron-Specific Based on Spiny Core-Shell Au/Cu_x_O@CeO_2_ Nanocubes. Bioelectrochemistry.

[13] Lobera E.S., Varela M.A., Jimenez R.L., Moreno R.B. (2023). MiRNA as Biomarker in Lung Cancer. Mol. Biol. Rep..

[14] Saasa V., Chibagidi R., Ipileng K., Feleni U. (2025). Advances in Cancer Detection: A Review on Electrochemical Technologies. Sens. Biosens. Res..

[15] Clark L., Lyons L. (1962). Glucose Enzyme Electrode. Ann. N. Y. Acad. Sci..

[16] Palchetti I., Mascini M. (2009). Biosensor Technology: A Brief History.

[17] Liu C., Lin P., Li S., Yao Y., Wang Z., Hu W.W. (2026). Toward Continuous and Non-Invasive Monitoring: A Scoping Review of in-Vitro Blood Glucose Devices from Electrochemistry to Optics and micro-System Integration. J. Mater. Chem. B.

[18] Feleni U., Morare R., Masunga G.S., Magwaza N., Saasa V., Madito M.J., Managa M. (2025). Recent Developments in Waterborne Pathogen Detection Technologies. Environ. Monit. Assess..

[19] Khalil I., Julkapli N.M., Yehye W.A., Basirun W.J., Bhargava S.K. (2016). Graphene–Gold Nanoparticles Hybrid—Synthesis, Functionalization, and Application in a Electrochemical and surface-Enhanced Raman Scattering Biosensor. Materials.

[20] Ipeleng K.E., Feleni U., Saasa V. (2025). Recent Developments on Salmonella and Listeria Monocytogenes Technologies: A Focus on Electrochemical Biosensing. Foods.

[21] Tan C., Zhao J., Sun P., Zheng W., Cui G. (2020). Gold Nanoparticle Decorated Polypyrrole/Graphene Oxide nanosheets as a Modified Electrode for Simultaneous Determination of Ascorbic, Dopamine and Uric Acid. New J. Chem..

[22] Wu Y., Deng P., Tian Y., Feng J., Xiao J., Li J., Liu J., Li G., He Q. (2020). Simultaneous and Sensitive Determination of Ascorbic Acid, Dopamine and Uric Acid via an Electrochemical Sensor Based on PVP-Graphene Composite. J. Nanobiotechnol..

[23] Joshi S., Kallappa S., Kumar P., Shukla S., Ghosh R. (2022). Simple Diagnosis of Cancer by Detecting CEA and CYFRA 21-1 in Saliva Using Electronic Sensors. Sci. Rep..

[24] Yang G., Li L., Lee W.B., Ng M.C. (2018). Structure of Graphene and Its Disorders: A Review. Sci. Technol. Adv. Mater..

[25] Ozkan-Ariksoysal D. (2022). Current Perspectives in Graphene Oxide-Based Electrochemical Biosensors for Cancer Diagnostics. Biosensors.

[26] Yu W., Sisi L., Haiyan Y., Jie L. (2020). Progress in the Functional Modification of Graphene/Graphene: A Review. RSC Adv..

[27] Botas C., Álvarez P., Blanco P., Granda M., Blanco C., Santamaría R., Romasanta L.J., Verdejo R., López-Manchado M.A., Menéndez R. (2013). Graphene Materials with Different Structures Prepared from the same Graphite by the Hummers and Brodie Methods. Carbon.

[28] Poh H.L., Šaněk F., Ambrosi A., Zhao G., Sofer Z., Pumera M. (2012). Graphenes Prepared by Staudenmaier, Hofmann and Hummers methods with Consequent Thermal Exfoliation Exhibit Very Different Properties. Nanoscale.

[29] Schmelter D., Hintze-Bruening H. (2016). Highly Ordered Graphene Oxide and Reduced Graphene Oxide Based Nanocomposites: Promise and Limits for Dynamic Impacts in Model Organic Coatings. ACS Appl. Mater. Interfaces.

[30] Zaaba N.I., Foo K.L., Hashim U., Tan S.J., Liu W., Voon C.H. (2017). Synthesis of Graphene Oxide Using Modified Hummers Method: Solvent. Procedia Eng..

[31] Kim J., Eum J., Kang J., Kwon O., Kim H., Kim D.W. (2021). Tuning the Hierarchical Pore Structure of Graphene Oxide through dual Thermal Activation for High-Performance Supercapacitor. Sci. Rep..

[32] Ambrosi A., Chua C.K., Bonanni A., Pumera M. (2014). Electrochemistry of Graphene and Related Materials. Chem. Rev..

[33] Shoja Y., Kermanpur A., Karimzadeh F. (2018). Diagnosis of EGFR Exon21 L858R Point Mutation as Lung Cancer by Electrochemical DNA Biosensor Based on Reduced Oxide/Functionalized Ordered Mesoporous/Ni-Oxytetracycline Metallopolymer Nanoparticles Modified Graphite Electrode. Biosens. Bioelectron..

[34] Xiao K., Wang K., Qin W., Hou Y., Lu W., Xu H., Wo Y., Cui D. (2017). Use of Quantum Dot Beads-Labeled Monoclonal Antibody to improve the Sensitivity of a Quantitative and Simultaneous Assay for Neuron Specific Enolase and carcinoembryonic Antigen. Talanta.

[35] Feng L., Qin W., Wang Y., Gu C., Li X., Chen J., Chen J., Qiao H., Yang M., Tian Z. (2023). Ti3C2Tx MXene/Graphene/AuNPs 3D Porous Composites for High and Fast Response Glucose Biosensing. Microchem. J..

[36] Zeng H., Xie Y., Liu T., Chu Z., Dempsey E., Jin W. (2024). Conductive Polymer Nanocomposites: Recent Advances in the construction of Electrochemical Biosensors. Sens. Diagn..

[37] Wei Y., Zhang Y., Chen J., Mao C., Jin B. (2020). An Electrochemiluminescence Biosensor for P53 Antibody Based On-MOF/GO Nanocomposite and Ag-DNA Amplification. Microchim. Acta.

[38] Sengupta J., Hussain C.M. (2023). Early Detection of Cancer Utilizing Biosensors Based on “Green’’: An Innovative and Sustainable Methodology for advancing Cancer Diagnosis. Trends Anal. Chem..

[39] Zhao D., Wang Y., Nie G. (2016). Electrochemical Immunosensor for the Carcinoembryonic Antigen on a Nanocomposite Consisting of Reduced Graphene Oxide, Gold Nanoparticles and Poly(Indole-6-Carboxylic Acid). Microchim. Acta.

[40] Shamsazar A., Soheili-Moghaddam M., Asadi A. (2024). A Novel Electrochemical Immunosensor Based on MWCNT/CuO for Effectively Detection of Carcinoembryonic (CEA). Microchem. J..

[41] Chiu N., Lin T., Kuo C. (2018). Highly Sensitive Carboxyl-Graphene Oxide-Based Surface Plasmon Immunosensor for the Detection of Lung Cancer for cytokeratin 19 Biomarker in Human Plasma. Sens. Actuators B Chem..

[42] Huang J., Lin H., Chen T., Chen C., Chang H., Chen C. (2018). Signal Amplified Gold Nanoparticles for Cancer Diagnosis On-Based Analytical Devices. ACS Sens..

[43] Chang L., Wu H., Chen R., Sun X., Yang Y., Huang C., Ding S., Liu C., Cheng W. (2023). Microporous PdCuB Nanotag-Based Electrochemical Aptasensor with Au@CuCl2 Nanowires Interface for Ultrasensitive Detection of PD-L1-Positive Exosomes in the Serum of Lung Cancer Patients. J. Nanobiotechnology.

[44] Bilal M., Bandyopadhyay S. (2025). Controlled Growth of Citrate-Stabilized Gold Nanoparticles Using a semi-Continuous Seed-Mediated Route. Discov. Nano.

[45] Li C., Chan M., Chang Y., Hsiao M. (2023). Gold Nanoparticles as a Biosensor for Cancer Biomarker. Molecules.

[46] Al Fatease A., Guo W., Umar A., Zhao C., Alhamhoom Y., Muhsinah A.B., Mahnashi M.H., Ansari Z.A. (2022). A Dual-Mode Electrochemical Aptasensor for the Detection of Mucin-1 Based on AuNPs-Magnetic Graphene Composite. Microchem. J..

[47] Su S., Sun H., Cao W., Chao J., Peng H., Zuo X., Yuwen L., Fan C., Wang L. (2016). Dual-Target Electrochemical Biosensing Based on DNA Structural on Gold Nanoparticle-Decorated MoS2 Nanosheets. ACS Appl. Mater. Interfaces.

[48] Wong A.C. (2017). Probing the Cellular Uptake of DNA Functionalized Gold. Ph.D. Thesis.

[49] Tomita M., Shimizu T., Matsuzaki Y., Hara M., Ayabe T., Onitsuka T. (2005). Prognostic Significance of Carcinoembryonic Antigen Level in pleural Lavage Fluid for Patients with Lung Adenocarcinoma. Ann. Thorac. Surg..

[50] Grunnet M., Sorensen J.B. (2012). Carcinoembryonic Antigen (CEA) as Tumor Marker in Lung Cancer. Lung Cancer.

[51] Jafari-Kashi A., Rafiee-Pour H., Shabani-Nooshabadi M. (2022). A New Strategy to Design Label-Free Electrochemical Biosensor for ultrasensitive Diagnosis of CYFRA 21–1 as a Biomarker for detection of Non-Small Cell Lung Cancer. Chemosphere.

[52] Buccheri G., Ferrigno D. (2003). Identifying Patients at Risk of Early Postoperative Recurrence of Lung Cancer: A New Use of the Old CEA Test. Ann. Thorac. Surg..

[53] Zhang G., Liu Z., Fan L., Guo Y. (2018). Electrochemical prostate specific antigen aptasensor based on hemin functionalized graphene-conjugated palladium nanocomposites. Microchim. Acta.

[54] Karnwal A., Kumar Sachan R.S., Devgon I., Devgon J., Pant G., Panchpuri M., Ahmad A., Alshammari M.B., Hossain K., Kumar G. (2024). Gold Nanoparticles in Nanobiotechnology: From Synthesis to Biosensing Applications. ACS Omega.

[55] Mahato K., Nagpal S., Shah M.A., Srivastava A., Maurya P.K., Roy S., Jaiswal A., Singh R., Chandra P. (2019). Gold Nanoparticle Surface Engineering Strategies and Their Applications in Biomedicine and Diagnostics. 3 Biotech.

[56] Patil T.P., Vibhute A.A., Vinu A., Pandey-Tiwari A. (2025). Nucleic Acid Conjugated Gold Nanoparticles for Biosensing Applications: A Review. World J. Microbiol. Biotechnol..

[57] Zhang P., Zhang X., Zhang S., Lu X., Li Q., Su Z., Wei G. (2013). One-Pot Green Synthesis, Characterizations, and Biosensor Application of Self-Assembled Reduced Graphene Oxide–Gold Nanoparticle Hybrid Membranes. J. Mater. Chem. B.

[58] Szuplewska A., Kulpińska D., Dybko A., Chudy M., Jastrzębska A.M., Olszyna A., Brzózka Z. (2020). Future Applications of MXenes in Biotechnology, Nanomedicine, and Sensors. Trends Biotechnol..

[59] Ibrahim B., Akere T.H., Dhumal P., Valsami-Jones E., Chakraborty S. (2025). Designing Safer Nanohybrids: Stability and Ecotoxicological Assessment of Graphene Oxide-Gold Nanoparticle Hybrids in Embryonic Zebrafish. Environ. Sci. Nano.

[60] Lebepe T.C., Oluwafemi O.S. (2022). Thermal and Medium Stability Study of Polyvidone-Modified Graphene Oxide-Coated Gold Nanorods with High Photothermal Efficiency. Nanomaterials.

[61] Al-Ani L.A., Yehye W.A., Kadir F.A., Hashim N.M., AlSaadi M.A., Julkapli N.M., Hsiao V.K.S. (2019). Hybrid Nanocomposite Curcumin-Capped Gold Nanoparticle-Reduced Graphene Oxide: Anti-Oxidant Potency and Selective Cancer Cytotoxicity. PLoS ONE.

[62] Szustakiewicz P., Kolsut N., Leniart A., Lewandowski W. (2019). Universal Method for Producing Reduced Graphene Oxide/Gold Nanoparticles Composites with Controlled Density of Grafting and Long-Term Stability. Nanomaterials.

[63] Feng R., Fan Y., Fang Y., Xia Y. (2023). Morphological Effects of Au Nanoparticles on Electrochemical Sensing Platforms for Nitrite Detection. Molecules.

[64] Rudin C.M., Brambilla E., Faivre-Finn C., Sage J. (2021). Small-Cell Lung Cancer. Nat. Rev. Dis. Prim..

[65] Dayon L., Cominetti O., Affolter M. (2022). Proteomics of Human Biological Fluids for Biomarker Discoveries: Technical Advances and Recent Applications. Expert Rev. Proteom..

[66] Corrie S.R., Coffey J.W., Islam J., Markey K.A., Kendall M. (2015). Blood, Sweat, and Tears: Developing Clinically Relevant Protein for Integrated Body Fluid Analysis. Analyst.

[67] Hotta T., Takifuji K., Yokoyama S., Matsuda K., Oku Y., Nasu T., Ieda J., Yamamoto N., Iwamoto H., Takei Y. (2014). Impact of the Post/Preoperative Serum CEA Ratio on the survival of Patients with Rectal Cancer. Surg. Today.

[68] Wang J., Bei J., Guo X., Ding Y., Chen T., Lu B., Wang Y., Du Y., Yao Y. (2022). Ultrasensitive Photoelectrochemical Immunosensor for Carcinoembryonic Antigen Detection Based on Pillar [5] Arene-Functionalized Au Nanoparticles and Hollow PANI Hybrid BiOBr Heterojunction. Biosens. Bioelectron..

[69] Holdenrieder S., Wehnl B., Hettwer K., Simon K., Uhlig S., Dayyani F. (2017). Carcinoembryonic Antigen and Cytokeratin-19 Fragments for assessment of Therapy Response in Non-Small Cell Lung Cancer: A Review and Meta-Analysis. Br. J. Cancer.

[70] Hao C., Zhang G., Zhang L. (2019). Serum CEA Levels in 49 Different Types of Cancer and Noncancer. Prog. Mol. Biol. Transl. Sci..

[71] Zhang X., Tan X., Wang P., Qin J. (2023). Application of Polypyrrole-Based Electrochemical Biosensor for the Early Diagnosis of Colorectal Cancer. Nanomaterials.

[72] Tomita M., Ayabe T., Chosa E., Nakamura K. (2015). Postoperative Serum CEA Level Is a More Significant Prognostic than Post/Preoperative Serum CEA Ratio in Non-Small Cell Patients. Asian Pac. J. Cancer Prev..

[73] Sawabata N., Kanzaki R., Sakamoto T., Kusumoto H., Kimura T., Nojiri T., Kawamura T., Susaki Y., Funaki S., Nakagiri T. (2014). Clinical Predictor of Pre-or Minimally Invasive Pulmonary: Possibility of Sub-Classification of Clinical T1a. Eur. J. Cardio-Thorac. Surg..

[74] Špringer T., Homola J. (2012). Biofunctionalized Gold Nanoparticles for SPR-Biosensor-Based Detection of CEA in Blood Plasma. Anal. Bioanal. Chem..

[75] Iwasaki A., Shirakusa T., Yoshinaga Y., Enatsu S., Yamamoto M. (2004). Evaluation of the Treatment of Non-Small Cell Lung Cancer with brain Metastasis and the Role of Risk Score as a Survival. Eur. J. Cardio-Thorac. Surg..

[76] Liu N., Xu Z., Morrin A., Luo X. (2019). Low Fouling Strategies for Electrochemical Biosensors Targeting Disease Biomarkers. Anal. Methods.

[77] Giovanella L., Imperiali M., Trimboli P. (2017). Role of Serum Cytokeratin 19 Fragment (Cyfra 21.1) as a Prognostic Biomarker in Patients with Differentiated Thyroid Cancer. Sci. Rep..

[78] Satoh H., Ishikawa H., Kurishima K., Yamashita Y.T., Ohtsuka M., Sekizawa K. (2002). Cut-off Levels of NSE to Differentiate SCLC from NSCLC. Oncol. Rep..

[79] Nakata B., Takashima T., Ogawa Y., Ishikawa T., Hirakawa K. (2004). Serum CYFRA 21-1 (Cytokeratin-19 Fragments) Is a Useful Tumour for Detecting Disease Relapse and Assessing Treatment in Breast Cancer. Br. J. Cancer.

[80] Brechot J.M., Chevret S., Nataf J., Le Gall C., Fretault J., Rochemaure J., Chastang C. (1997). Diagnostic and Prognostic Value of Cyfra 21-1 Compared with Other Markers in Patients with Non-Small Cell Lung Cancer: A Study of 116 Patients. Eur. J. Cancer.

[81] Zhang Z., Li Q., Du X., Liu M. (2020). Application of Electrochemical Biosensors in Tumor Cell Detection. Thorac. Cancer.

[82] Pan L., Kuo S., Lin T., Lin C., Fang P., Yang H. (2017). An Electrochemical Biosensor to Simultaneously Detect VEGF and PSA for Early Prostate Cancer Diagnosis Based on Graphene/SsDNA/PLLA Nanoparticles. Biosens. Bioelectron..

[83] Kim P., Choi M.Y., Lee Y., Lee K., Choi J. (2025). Multiplexed Optical Nanobiosensing Technologies for Disease Detection. Biosensors.

[84] Wang Y., Wang H., Bai Y., Zhao G., Zhang N., Zhang Y., Wang Y., Chi H. (2023). MoS2@Au as Label for Sensitive Sandwich-Type Immunoassay of Neuron-Specific Enolase. Chemosensors.

[85] Genet S.A., Visser E., van den Borne B.E., Youssef-El Soud M., Belderbos H.N., Stege G., de Saegher M.E., Eduati F., Broeren M.A., van Dongen J. (2020). Correction of the NSE Concentration in Hemolyzed Serum Samples Its Diagnostic Accuracy in Small-Cell Lung Cancer. Oncotarget.

[86] Mastroianni A., Panella R., Morelli D. (2020). Invisible Hemolysis in Serum Samples Interferes in NSE. Tumori J..

[87] Wang B., Liang T., Li J., Yu H., Chu X. (2017). Fabrication of Immunosensor Based on Au-Silica Nanocomposite for neuron-Specific Enolase Detection. Int. J. Electrochem. Sci..

[88] Isgrò M.A., Bottoni P., Scatena R. (2015). Neuron-specific enolase as a biomarker: Biochemical and clinical aspects. Adv. Exp. Med. Biol..

[89] Xing Z., Zhang S., Wang H., Ma H., Wu D., Fan D., Ren X., Wei Q., Ju H. (2022). Addressable Label-Free Photoelectric Sensor Array with self-Calibration for Detection of Neuron Specific Enolase. Anal. Chem..

[90] Wani J.A., Majid S., Imtiyaz Z., Rehman M.U., Alsaffar R.M., Shah N.N., Alshehri S., Ghoneim M.M., Imam S.S. (2022). MiRNAs in Lung Cancer: Diagnostic, Prognostic, and Therapeutic. Diagnostics.

[91] Zhao W., Zhao J., Zhang L., Xu Q., Zhao Y., Shi X., Xu A. (2015). Serum MiR-21 Level: A Potential Diagnostic and Prognostic for Non-Small Cell Lung Cancer. Int. J. Clin. Exp. Med..

[92] Yang M., Shen H., Qiu C., Ni Y., Wang L., Dong W., Liao Y., Du J. (2013). High Expression of MiR-21 and MiR-155 Predicts Recurrence and unfavourable Survival in Non-Small Cell Lung Cancer. Eur. J. Cancer.

[93] Shao C., Yang F., Qin Z., Jing X., Shu Y., Shen H. (2019). The Value of MiR-155 as a Biomarker for the Diagnosis and prognosis of Lung Cancer: A Systematic Review with Meta-Analysis. BMC Cancer.

[94] Goyal A., Afzal M., Goyal K., Ballal S., Sharma G.C., Kavitha V., Maharana L., Devi A., Rana M., Kumar K.B. (2025). MiR-210: A Non-Invasive Biomarker for Hypoxia-Driven Lung Cancer and Therapy. Clin. Chim. Acta.

[95] Grimolizzi F., Monaco F., Leoni F., Bracci M., Staffolani S., Bersaglieri C., Gaetani S., Valentino M., Amati M., Rubini C. (2017). Exosomal MiR-126 as a Circulating Biomarker in Non-Small-Cell Lung Regulating Cancer Progression. Sci. Rep..

[96] Jiang Y., Huang J., Wu J., Eda S. (2022). A Rapid, Sensitive, and Simple-to-Use Biosensor for on-Site of Attomolar Level MicroRNA Biomarkers from Serum Vesicles. Sens. Actuators B Chem..

[97] Ren R., Bi Q., Yuan R., Xiang Y. (2020). An efficient, label-free and sensitive electrochemical microRNA sensor based on target-initiated catalytic hairpin assembly of trivalent DNAzyme junctions. Sens. Actuators B Chem..

[98] Hossein Zadeh R., Hossein Zadeh R., Hajimoradi M., Islampanah M., Zarimeidani F., Rahmati R., Ahmadinia M., Bahrami N., Mohamadnia A., Shafaghi S. (2026). Identification of diagnostic and prognostic biomarkers in lung adenocarcinoma through integrated bioinformatics analysis and real time PCR validation. Sci. Rep..

[99] Zhou B., Hu S., Chen W., Xiang S., Xue Y., Zhang W., Yang R. (2025). Visualized chemiluminescence immunoassay for simultaneous detection of multiple lung cancer markers based on spatially resolved microfluidic paper chip device. Talanta.

[100] Wang L., Wang D., Zheng G., Yang Y., Du L., Dong Z., Zhang X., Wang C. (2016). Clinical evaluation and therapeutic monitoring value of serum tumor markers in lung cancer. Int. J. Biol. Markers..

[101] Izawa A., Hara Y., Horita N., Muraoka S., Kaneko M., Kaneko A., Somekawa K., Hirata M., Otsu Y., Matsumoto H. (2024). Improved diagnostic accuracy with three lung tumor markers compared to six-marker panel. Transl. Lung Cancer Res..

[102] Zhang Q., Fu Y., Xiao K., Du C., Zhang X., Chen J. (2021). Sensitive dual-mode biosensors for CYFRA21-1 assay based on the dual-signaling electrochemical ratiometric strategy and “on–off–on” PEC method. Anal. Chem..

[103] Cao L., Zhang W., Lu S., Guo C., Wang P., Zhang D., Ma W. (2021). A Label-Free Electrochemical Immunosensor for CEA Detection on a Novel Signal Amplification Platform of Cu2S/Pd/CuO Nanocomposites. Front. Bioeng. Biotechnol..

[104] Karaman C., Bölükbaşı Ö.S., Yola B.B., Karaman O., Atar N., Yola M.L. (2022). Electrochemical Neuron-Specific Enolase (NSE) Immunosensor Based on CoFe_2_O_4_@Ag Nanocomposite and AuNPs@MoS_2_/RGO. Anal. Chim. Acta.

[105] Hianik T. (2018). Aptamer-Based Biosensors. Encyclopedia of Interfacial Chemistry.

[106] Wang Y., Luo J., Liu J., Sun S., Xiong Y., Ma Y., Yan S., Yang Y., Yin H., Cai X. (2019). Label-Free Microfluidic Paper-Based Electrochemical Aptasensor for Ultrasensitive and Simultaneous Multiplexed Detection of Cancer Biomarkers. Biosens. Bioelectron..

[107] Wang X., Li X., Xie Y., Fang H., Cui X., Jiao J., Zhang Y. (2025). An electrochemical aptasensor based on synergistic effect of duplex-specific nuclease and nanoporous gold for ultrasensitive detection of prostate-specific antigen. Bioelectrochemistry.

[108] Yunussova N., Tilegen M., Pham T.T., Kanayeva D. (2024). Rapid Detection of Carcinoembryonic Antigen by Means of an Electrochemical Aptasensor. iScience.

[109] Xu L., Liu Z., Lei S., Huang D., Zou L., Ye B. (2019). A Sandwich-Type Electrochemical Aptasensor for the Carcinoembryonic Antigen via Biocatalytic Precipitation Amplification and by Using Gold Nanoparticle Composites. Microchim. Acta.

[110] Mikaeeli Kangarshahi B., Naghib S.M., Rabiee N. (2024). DNA/RNA-Based Electrochemical Nanobiosensors for Early Detection of Cancers. Crit. Rev. Clin. Lab. Sci..

[111] Zhang L., Su W., Liu S., Huang C., Ghalandari B., Divsalar A., Ding X. (2022). Recent Progresses in Electrochemical DNA Biosensors for MicroRNA Detection. Phenomics.

[112] Zhu C.S., Zhu L., Tan D.A., Qiu X.Y., Liu C.Y., Xie S.S., Zhu L.Y. (2019). Avenues Toward MicroRNA Detection In Vitro: A Review of Technical Advances and Challenges. Comput. Struct. Biotechnol. J..

[113] Cordeiro M., Carlos F.F., Pedrosa P., Lopez A., Baptista P.V. (2016). Gold Nanoparticles for Diagnostics: Advances towards Points of Care. Diagnostics.

[114] Saidur M.R., Aziz A.R.A., Basirun W.J. (2017). Recent Advances in DNA-Based Electrochemical Biosensors for Heavy Metal Ion Detection: A Review. Biosens. Bioelectron..

[115] Sheervalilou R., Shahraki O., Hasanifard L., Shirvaliloo M., Mehranfar S., Lotfi H., Pilehvar-Soltanahmadi Y., Bahmanpour Z., Zadeh S.S., Nazarlou Z. (2020). Electrochemical Nano-Biosensors as Novel Approach for the detection of Lung Cancer-Related MicroRNAs. Curr. Mol. Med..

[116] Zhang H., Fan M., Jiang J., Shen Q., Cai C., Shen J. (2019). Sensitive Electrochemical Biosensor for MicroRNAs Based on Duplex-Specific Nuclease-Assisted Target Recycling Followed with Gold Nanoparticles and Enzymatic Signal Amplification. Anal. Chim. Acta.

[117] Jian Z., Zhang X., Bi S. (2022). Two-dimensional quantum dot-based electrochemical biosensors. Biosensors.

[118] Asadzadeh-Firouzabadi A., Zare H.R. (2018). Preparation and Application of AgNPs/SWCNTs Nanohybrid as an Electroactive Label for Sensitive Detection of MiRNA Related to Lung Cancer. Sens. Actuators B Chem..

[119] Mohammadpour-Haratbar A., Boraei S.B.A., Zare Y., Rhee K.Y., Park S.J. (2023). Graphene-based electrochemical biosensors for breast cancer detection. Biosensors.

[120] Pastorino U., Boeri M., Sestini S., Sabia F., Milanese G., Silva M., Suatoni P., Verri C., Cantarutti A., Sverzellati N. (2022). Baseline Computed Tomography Screening and Blood MicroRNA Lung Cancer Risk and Define Adequate Intervals in the BioMILD Trial. Ann. Oncol..

[121] Torul H., Yarali E., Eksin E., Ganguly A., Benson J., Tamer U., Papakonstantinou P., Erdem A. (2021). Based Electrochemical Biosensors for Voltammetric Detection of miRNA Biomarkers Using Reduced Graphene Oxide or MoS_2_ Decorated with Gold Nanoparticle Electrodes. Biosensors.

[122] Pothipor C., Aroonyadet N., Bamrungsap S., Jakmunee J., Ounnunkad K. (2021). A Highly Sensitive Electrochemical MicroRNA-21 Biosensor Based on Intercalating Methylene Blue Signal Amplification and a Highly Dispersed Gold Nanoparticles/Graphene/Polypyrrole Composite. Analyst.

[123] Pothipor C., Jakmunee J., Bamrungsap S., Ounnunkad K. (2021). An Electrochemical Biosensor for Simultaneous Detection of Breast Cancer Clinically Related MicroRNAs Based on a Gold Nanoparticles/Graphene Quantum Dots/Graphene Oxide Film. Analyst.

[124] Cui L., Wang M., Sun B., Ai S., Wang S., Zhang C. (2019). Substrate-Free and Label-Free Electrocatalysis-Assisted biosensor for Sensitive Detection of MicroRNA in Lung Cancer Cells. Chem. Commun..

[125] Akbarnia A., Zare H.R., Moshtaghioun S.M., Benvidi A. (2019). Highly Selective Sensing and Measurement of MicroRNA-541 based on Its Sequence-Specific Digestion by the Restriction Enzyme Hinf1. Colloids Surf. B Biointerfaces.

[126] Lusi E.A., Passamano M., Guarascio P., Scarpa A., Schiavo L. (2009). Innovative Electrochemical Approach for an Early Detection of MicroRNAs. Anal. Chem..

[127] Wei H., Gao L., Fan K., Liu J., He J., Qu X., Dong S., Wang E., Yan X. (2021). Nanozymes: A Clear Definition with Fuzzy Edges. Nano Today.

[128] Thamilselvan A., Kim M. (2024). Il Recent Advances on Nanozyme-Based Electrochemical Biosensors for Cancer Biomarker Detection. TrAC-Trends Anal. Chem..

[129] Mahmudunnabi R.G., Farhana F.Z., Kashaninejad N., Firoz S.H., Shim Y., Shiddiky M.J. (2020). Nanozyme-Based Electrochemical Biosensors for Disease Biomarker. Analyst.

[130] Tang G., He J., Liu J., Yan X., Fan K. (2021). Nanozyme for Tumor Therapy: Surface Modification Matters. Exploration.

[131] Tian Q., Li S., Tang Z., Zhang Z., Du D., Zhang X., Niu X., Lin Y. (2025). Nanozyme-Enabled Biomedical Diagnosis: Advances, Trends, And. Adv. Healthc. Mater..

[132] Pushpalatha C., Sowmya S.V., Augustine D., Kumar C., Gayathri V.S., Shakir A., Prabhu T.N., Sandhya K.V., Patil S. (2022). Antibacterial Nanozymes: An Emerging Innovative Approach to Oral Health Management. Top. Catal..

[133] Jin G.H., Ko E., Kim M.K., Tran V.-K., Son S.E., Geng Y., Hur W., Seong G.H. (2018). Graphene Oxide-Gold Nanozyme for Highly Sensitive Electrochemical of Hydrogen Peroxide. Sens. Actuators B Chem..

[134] Adegoke O., Zolotovskaya S., Abdolvand A., Nic Daeid N. (2021). Rapid and Highly Selective Colorimetric Detection of Nitrite based on the Catalytic-Enhanced Reaction of Mimetic Au-CeO2 Nanoparticle-Graphene Oxide Hybrid Nanozyme. Talanta.

[135] Chen G., Qin Y., Jiao L., Huang J., Wu Y., Hu L., Gu W., Xu D., Zhu C. (2021). Nanozyme-Activated Synergistic Amplification for Ultrasensitive Photoelectrochemical Immunoassay. Anal. Chem..

[136] Ko E., Tran V.-K., Son S.E., Hur W., Choi H., Seong G.H. (2019). Characterization of Au@PtNP/GO Nanozyme and Its Application to Electrochemical Microfluidic Devices for Quantification of Hydrogen Peroxide. Sens. Actuators B Chem..

[137] Yuan B., Xu C., Liu L., Shi Y., Li S., Zhang R., Zhang D. (2014). Polyethylenimine-Bridged Graphene Oxide–Gold Film on Glassy Carbon Electrode and Its Electrocatalytic Activity toward Nitrite and Hydrogen Peroxide. Sens. Actuators B Chem..

[138] Chang H., Wang X., Shiu K.-K., Zhu Y., Wang J., Li Q., Chen B., Jiang H. (2013). Layer-by-Layer Assembly of Graphene, Au and Poly(Toluidine Blue O) Films Sensor for Evaluation of Oxidative Stress of Tumor Cells Elicited by Hydrogen Peroxide. Biosens. Bioelectron..

[139] Tao Y., Lin Y., Huang Z., Ren J., Qu X. (2013). Incorporating Graphene Oxide and Gold Nanoclusters: A Synergistic Catalyst with Surprisingly High Peroxidase-like Activity over a Broad PH Range and Its Application for Cancer Cell Detection. Adv. Mater..

[140] Li Z., Li H., Geng X., Dai G., Chu Z., Luo F., Zhang F., Wang Q. (2022). Sandwich-Type Electrochemical Sensor for Mucin-1 Detection based on a Cysteine–Histidine–Cu@Cuprous Oxide Nanozyme. ACS Appl. Nano Mater..

[141] Fu L., Liu X., Cao J., Li H., Xie A., Liu Y. (2024). Recent Advance in Electrochemical Immunosensors for Lung Cancer Biomarkers Sensing. Rev. Anal. Chem..

[142] Crapnell R.D., Dempsey-Hibbert N.C., Peeters M., Tridente A., Banks C.E. (2020). Molecularly Imprinted Polymer Based Electrochemical Biosensors: Overcoming the Challenges of Detecting Vital Biomarkers and speeding up Diagnosis. Talanta Open.

[143] Tshemese Z., Masikane S.C., Mlowe S., Revaprasadu N. (2018). Progress in Green Solvents for the Stabilisation of Nanomaterials: Imidazolium Based Ionic Liquids. Recent Advances in Ionic Liquids.

[144] Shumbula N.P., Nkabinde S.S., Ndala Z.B., Mpelane S., Shumbula M.P., Mdluli P.S., Njengele-Tetyana Z., Tetyana P., Hlatshwayo T., Mlambo M. (2022). Evaluating the antimicrobial activity and cytotoxicity of polydopamine capped silver and silver/polydopamine core-shell nanocomposites. Arab. J. Chem..

[145] Zhu Q., Yang Y., Gao H., Xu L., Wang S. (2022). Bioinspired superwettable electrodes towards electrochemical biosensing. Chem. Sci..

[146] Hu Y., Liang B., Fang L., Ma G., Yang G., Zhu Q., Chen S., Ye X. (2016). Antifouling zwitterionic coating via electrochemically mediated atom transfer radical polymerization on enzyme-based glucose sensors for long-time stability in 37 C serum. Langmuir.

[147] Yokus M.A., Songkakul T., Pozdin V.A., Bozkurt A., Daniele M.A. (2020). And Wearable Multiplexed Biosensor System toward Continuous monitoring of Metabolites. Biosens. Bioelectron..

[148] Saasa V., Gumede T., Msweli N.T. (2026). Flexible Polymeric Materials and Wearable Biosensors for Smart Mellitus Diagnostics and Monitoring. iScience.

[149] Sun A.C., Hall D.A. (2019). Point-of-Care Smartphone-Based Electrochemical Biosensing. Electroanalysis.

[150] Danvirutai P., Ekpanyapong M., Tuantranont A., Bohez E., Anutrakulchai S., Wisitsoraat A., Srichan C. (2020). Ultra-Sensitive and Label-Free Neutrophil Gelatinase-Associated Electrochemical Sensor Using Gold Nanoparticles 3D Graphene Foam towards Acute Kidney Injury Detection. Sens. Biosens. Res..

[151] Ng S., Masarone S., Watson D., Barnes M.R. (2023). The Benefits and Pitfalls of Machine Learning for Biomarker Discovery. Cell Tissue Res..

[152] Chakraverty R., Debnath T. (2026). Nanotheragnostics for Personalized Care. Biomedical Theragnostics Nanoplatforms.

